# Spinal cord stimulation in non-reconstructable critical limb ischemia: a retrospective study of 71 cases

**DOI:** 10.1007/s00701-022-05448-8

**Published:** 2023-01-04

**Authors:** G. S. Piedade, J. Vesper, D. Reichstein, A. K. Dauphin, S. Damirchi

**Affiliations:** 1 Department of Neurosurgery, Helios Universitätsklinikum Wuppertal, Universität Witten/Herdecke, Heusnerstr. 40, 42283 Wuppertal, Germany; 2 Department of Stereotaxy and Functional Neurosurgery, Rheinische Friedrich-Wilhelms-Universität Bonn, Bonn, Germany; 3 Department of Neurosurgery, Heinrich-Heine-Universität Düsseldorf, Düsseldorf, Germany; 4 Department of Functional Neurosurgery and Stereotaxy, Heinrich-Heine-Universität Düsseldorf, Düsseldorf, Germany; 5 Department of Vascular Surgery, Evangelisches Krankenhaus Herne, Herne, Germany

**Keywords:** Spinal cord stimulation, Peripheral arterial disease, Critical limb ischemia, Neuromodulation

## Abstract

**Background:**

Spinal cord stimulation (SCS) is a therapeutic option for patients with a peripheral arterial disease with critical limb ischemia (CLI) and consequent ischemic rest pain. Neuromodulation is chosen when vascular reconstruction is not possible or failed. Data about the effect of SCS over limb salvage rates are dissonant.

**Method:**

We report on a retrospective cohort of CLI patients who were implanted with SCS systems between July 2010 and December 2013 in a single center. Major amputation, postoperative complications, and death were recorded.

**Results:**

Seventy-two CLI patients underwent SCS implantation, with 35 of them classified as non-reconstructable and 37 with previous but failed or only partially successful vascular procedures. A total of 21 subjects were at Fontaine’s stage III (29.2%), and the remaining 51 were at stage IV (70.8%). In total, 26.4% of the patients had diabetes (*n* = 19), two of them at Fontaine’s stage III. The mean follow-up was 17.1 ± 10.5 months. At the last follow-up, 59.2% of all patients (42/71), 85.7% of Fontaine’s stage III (18/21), 48.0% of Fontaine’s stage IV (24/50), and 52.6% of diabetic patients (10/19) were alive without major amputation. The probability of limb survival at 12 months was 72% for all patients, 94% for Fontaine’s stage III, 62% for Fontaine’s stage IV, and 61% for diabetic patients. The probability of survival at 12 months for patients who underwent major limb amputation (*n* = 25) was 86% with a mean survival time of 31.03 ± 4.63 months.

**Conclusions:**

Non-reconstructable CLI patients treated with SCS can achieve meaningful clinical outcomes with few procedure-related complications. The therapy may be more beneficial in patients classified as Fontaine’s Stage III.

## Introduction

Across the globe, peripheral arterial disease (PAD) is estimated to affect more than 200 million people, with a growth rate of 23.5% between 2000 and 2010. In high-income countries, prevalence is in the range of 3 to 4% for men and women aged 30 to 40 years and increases to around 10 to 20% in those over 65 years [5]. The Inter-Society Consensus for the Management of Peripheral Arterial Disease (TASC II) highlighted that 1 to 3% of PAD patients will develop critical limb ischemia (CLI), diagnosed by the present of ischemic rest pain associated with major amputations and that the need for major amputation in diabetic patients may be even higher at a rate of 5 to 10 times. Patients with CLI undergoing primary treatment with vascular reconstruction—surgical or endovascular—have a high risk of limb amputation (30%) or mortality within 1 year (25%) [9]. In CLI cases where vascular reconstruction is not possible or fails, spinal cord stimulation (SCS) should be explored.

Spinal cord stimulation is one of the secondary non-reconstructive treatment options currently available to CLI patients. Although the therapy was first introduced in the 1960s for the treatment of pain, Cook et al. first used SCS in PAD in 1976. They found an improvement in the healing of ulcers as well as in pain relief [3, 14]. In 2013, the Cochrane Collaboration reviewed 6 controlled studies, including a total of 444 patients, comparing SCS with conservative medical treatment in patients with non-reconstructable chronic CLI. The meta-analysis found that patients treated with SCS required less analgesia and had a significantly higher limb salvage rate after 12 months than patients who only had conservative medical treatment [14]. The mechanisms of action of SCS in vascular disease are not yet fully understood and are thought to be complex and multifaceted. Recent research suggests that SCS induces vasodilation in the peripheral microcirculatory system by a combination of antidromic activation of sensory fibers and sympathetic outflow reduction [15].

There are few studies reporting experience with SCS for PAD, and data about the effect of SCS over limb salvage rates are dissonant. We present a large retrospective cohort of CLI patients implanted with an SCS system in a single center**.**

## Material and methods

This retrospective study analyzed patients with CLI diagnosed between May 2010 and June 2014 in the Department of Vascular Surgery at the Evangelisches Krankenhaus Herne, Germany, and included those who underwent implantation of an SCS system. All patients were adults with the diagnosis of CLI according to Fontaine’s classification of PAD stage III (ischaemic rest pain) or IV (ulceration or gangrene) [4, 7]. The patients were also candidates for amputation.

The implantation of the SCS system in each patient was carried out by one of three vascular surgeons. Subjects with active infection or life expectancy shorter than 1 year were excluded. A preoperative stimulation trial was not performed. An octopolar SCS lead of St. Jude Medical was advanced to thoracolumbar spine T11-L1, and the implantable pulse generator (IPG) Genesis™ was used in 66 patients, EonC™ in 5 patients. Test stimulation was applied to ensure that the patient felt pleasant paresthesia in the target limb/foot and the lead repositioned if necessary. Patients were discharged from the hospital 5 days after surgery. Follow-up was performed for all patients immediately after the SCS system implantation, 6 weeks later, and thereafter every 3 months. During each follow-up, a clinical vascular examination and ultrasound were carried out in order to detect any proximal vascular disease. If necessary, a CT angiography was performed. Adjustments to stimulation were made according to clinical need.

Anonymized data related to demographics, Fontaine’s classification, diabetes and hypertension comorbidity, smoking history, previous vascular interventions, SCS system implantation details, postoperative complications, death, and amputation outcomes were retrospectively gathered from the patients’ medical records. The patient’s last follow-up was defined as the date of their last clinical follow-up if they were alive at the time of the study or the date of death.

Continuous variables were expressed as the mean ± standard deviation, and categorical variables were expressed as frequency and percentage. Cumulative event rates were analyzed using survival methods. Comparisons of the resulting Kaplan–Meier curves were carried out using log-rank tests.

This study received approval from the Ethics Committee of the Medical Faculty of the Heinrich-Heine University Düsseldorf (4964) and from the Ethics Committee of the Medical Board of Westfalen-Lippe (2015–260-b-S).

## Results

**Fig. 1:**
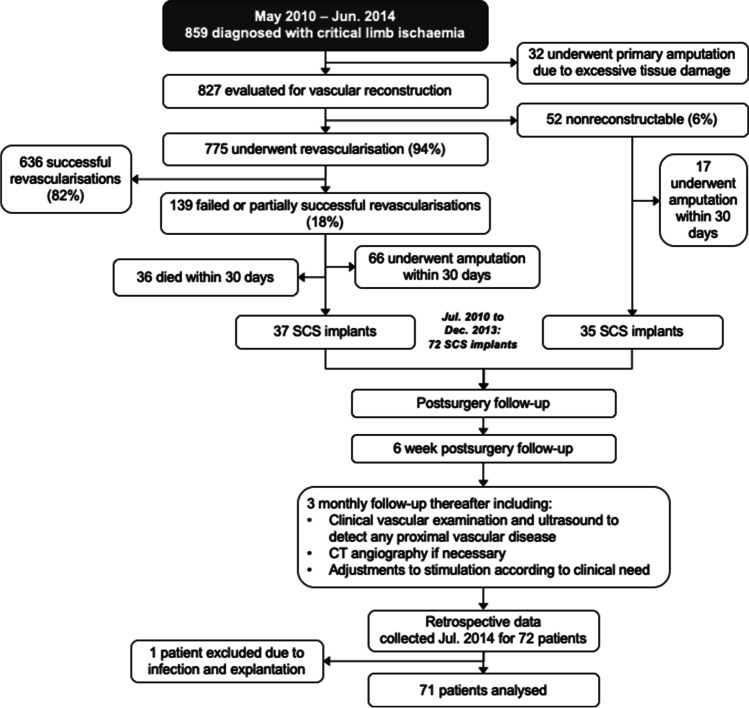
Flowchart illustrating patient pathways from diagnosis of critical limb ischemia (CLI)

Hospital records showed that 859 patients were diagnosed with CLI. Patients with non-reconstructable status or with failed or incomplete revascularization were candidates for spinal cord stimulation accordingly to Fig. 1. After the exclusion of subjects who underwent amputation shortly after diagnosis or died, a total of 72 patients (9%) were implanted with an SCS system. The implantation procedures occurred between July 2010 and December 2013. The baseline characteristics of this group are summarized in Table 1.

**Table 1:** Baseline characteristics of patients (*n* = 72)

Variable	*n* (%)
Sex	
Male	41 (56.9%)
Female	31 (43.1%)
Age	
Mean (years)	71.3 ± 10.9
Range (years)	46 to 93
Critical limb ischemia classification	
Fontaine’s stage III	21 (29.2%)
Fontaine’s stage IV	51 (70.8%)
Risk factors	
Diabetes	19 (26.4%)
History of smoking	51 (70.8%)
Hypertension	38 (52.8%)
Previous vascular intervention	
More than 2 previous vascular procedures	37 (51.4%)
No previous vascular procedures (non-reconstructable)	35 (48.6%)
Contralateral major amputation prior to spinal cord stimulation	6 (8.3%)

All patients (*n* = 72) were implanted with an octopolar SCS lead. One patient (1.4%) experienced an early infection and had the IPG removed. Shortly afterwards, the patient suffered a myocardial infarction and died before stimulation could start. We excluded this patient from subsequent statistical analysis. All other complications were minor. A total of 3 subjects (4.2%) experienced lead dislocation and required revision after 6, 14, and 19 months of therapy; all revisions were successful. Two patients (2.8%) had their IPGs replaced due to battery depletion shortly before stimulation was completed 1 year.

**Fig. 2:**
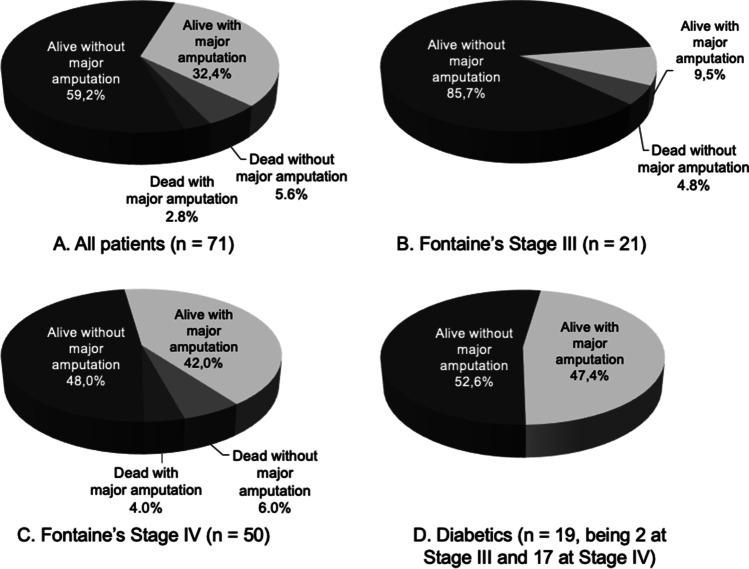
Status of patients at the last follow-up (last clinical follow-up or death) for all patients, Fontaine’s Stage III, Fontaine’s Stage IV, and diabetic patients

Follow-up duration (defined as the time from SCS implantation to last follow-up or date of death) ranged from 1.6 to 39.6 months with a mean of 17.1 ± 10.5 months. Data collection for the study stopped in June 2017, meaning that many subjects continued presenting to later follow-up appointments after this date. At the last follow-up, 42 patients (59.2%) were alive without major amputation; 23 (32.4%) were alive with major amputation; 4 subjects (5.6%) were dead without major amputation, and 2 (2.8%) were dead with major amputation (Fig. 2). The proportion of patients alive without major amputation was higher in Fontaine’s stage III patients at 85.7% (18/21) and lower in Fontaine’s stage IV patients at 48.0% (24/50). In the diabetic subgroup (*n* = 19), all patients were alive. Importantly, all but two diabetic patients were at Fontaine’s stage IV. Ten diabetic patients (52.6%) were alive without major amputation, and 9 (47.4%) were alive with major amputation.

**Fig. 3:**
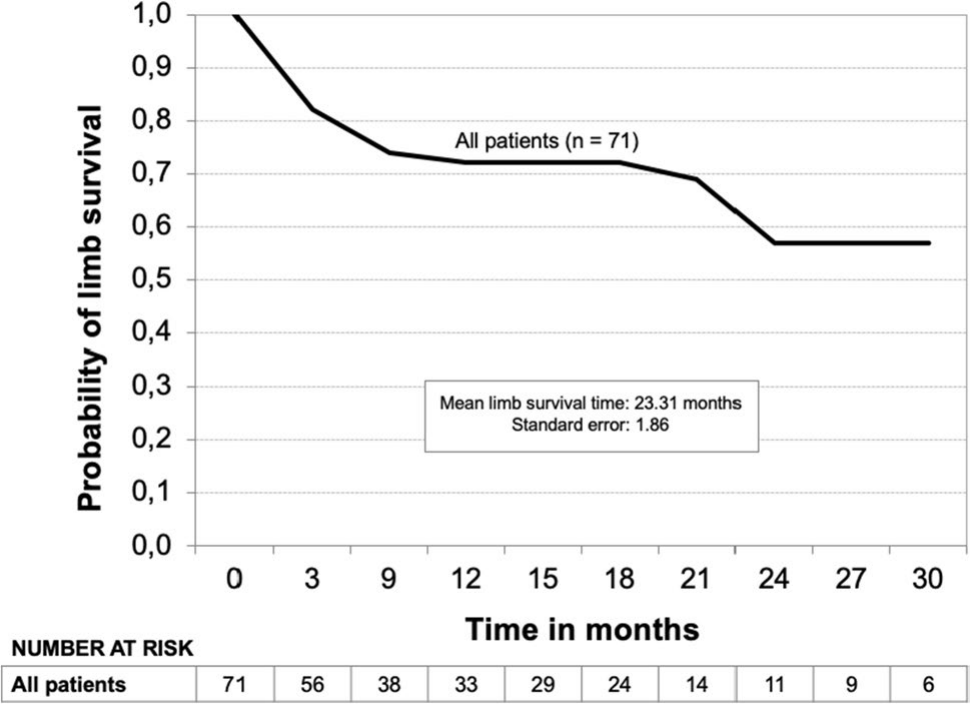
Kaplan–Meier limb survival curve for all patients (*n* = 71)

**Fig. 4:**
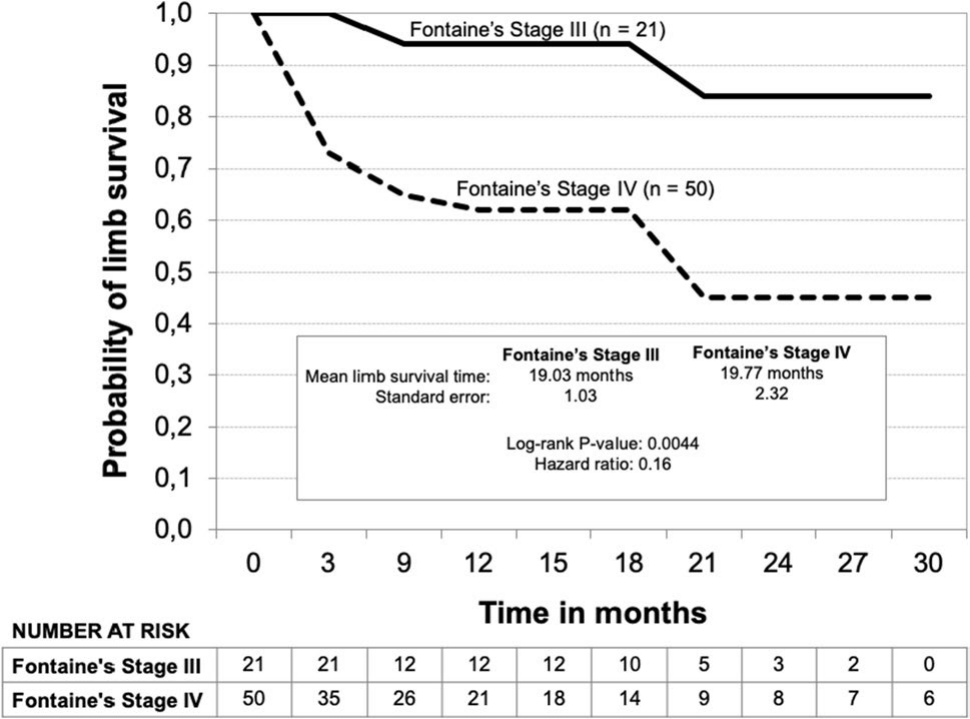
Kaplan–Meier limb survival curve for Fontaine’s Stage III (*n* = 21) and Fontaine’s Stage IV patients (*n* = 50)

**Fig. 5:**
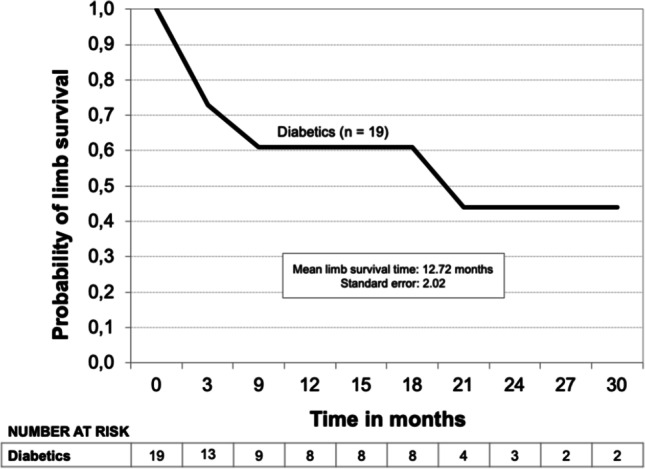
Kaplan–Meier limb survival curve for diabetic patients (*n* = 19)

**Fig. 6:**
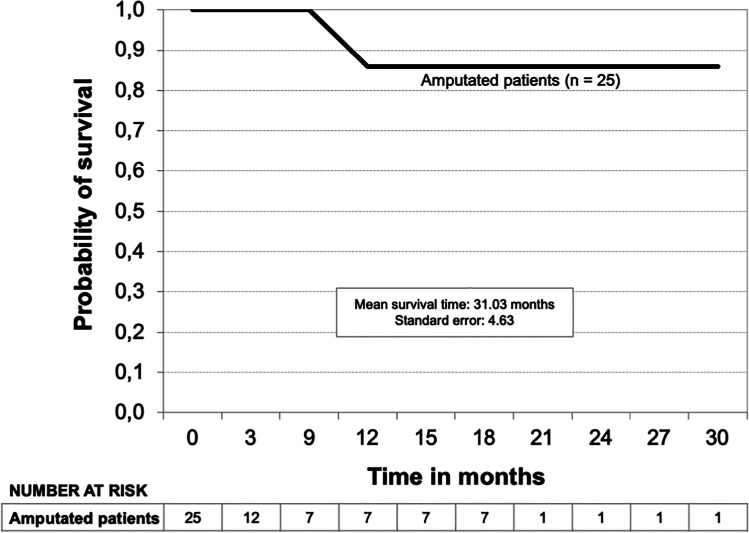
Kaplan–Meier survival curve for patients who underwent major limb amputation (*n* = 25)

Kaplan–Meier limb survival curves for all patients and the subgroups Fontaine’s stage III, Fontaine’s stage IV, and diabetic patients are shown in Figs. 3, 4, and 5. The probability of limb survival for all patients (*n* = 71) was 72% at 12 months with a mean limb survival time of 23.3 ± 1.86 months. The probability of limb survival in Fontaine’s stage III (*n* = 21) and Fontaine’s stage IV (*n* = 50) patients at 12 months was 94% and 62%, respectively (log-rank *P*-value = 0.0044; hazard ratio 0.16; reference group Fontaine’s stage IV). Mean limb survival time was 19.0 ± 1.03 months and 19.8 ± 2.32 months, respectively. In diabetic patients (*n* = 19), the probability of limb survival was 61% at 12 months with a mean limb survival time of 12.7 ± 2.02 months. A Kaplan–Meier survival curve was also developed for the subgroup of patients who underwent major limb amputation (*n* = 25) (Fig. 6). Within this subgroup, 23 patients (92%) were classified as Fontaine’s stage IV, and 2 (8%) were classified as Fontaine’s stage III. The probability of survival was 86% at both 12 and 24 months, and the mean survival time was 31.0 ± 4.63 months. Two patients within this group died (8%). Both patients were classified as Fontaine’s stage IV.

## Discussion

We found that the probability of limb survival at 12 months after SCS implantation was markedly higher in Fontaine’s stage III compared with Fontaine’s stage IV (94% vs. 62%), with major amputation occurring more slowly in Fontaine’s stage III (log-rank *P*-value = 0.0044; hazard ratio 0.16; reference group Fontaine’s stage IV). Using the data of this study, it is not possible to affirm that SCS is more advantageous earlier in stage III, and the worse outcomes in stage IV may be the consequence of more advanced disease.

The 2013 Cochrane Library meta-analysis on SCS for the treatment of non-reconstructable chronic CLI found that the pooled probability of limb survival at 12 months for CLI patients treated with SCS was 71% [14]. This value is very close to the 72% obtained in the present analysis for all patients. It should be noted, however, that in the Cochrane meta-analysis did not inform the proportion of patients in Fontaine’s stages III and IV. Gersbach et al. reported in 2007 a very similar cohort, this time with a similar proportion of patients in Fontaine’s stages III and IV and even with a longer follow-up. Results for limb salvage were similar but still slightly better and documented as well the sustained positive effect of SCS beyond the first year of treatment, with infrequent major amputations after 24 months of therapy [6].

We based our standard patient selection on the clinical, ultrasound, and angiographic findings, which ultimately determine the diagnosis of non-reconstructable chronic CLI. Other studies have shown that the probability of limb survival at 12 months after SCS implantation in CLI patients improves when patients are selected on the basis of transcutaneous oxygen pressure (TcpO_2_) measurements in the affected limb or a combination of TcpO_2_ measurements in the affected limb and a positive trial stimulation. The probability of limb survival was approximately 77% in the ESES study for patients with intermediate baseline TcpO_2_ [13]. In the SCS-EPOS study, it was 78% for the group of patients with TcpO_2_ below 30 mmHg at baseline and a positive stimulation trial and patients with TcpO_2_ below 10 mmHg at baseline rising to at least 20 mmHg after a positive stimulation trial (SCS-match group), while patients treated without SCS had a much lower probability of limb survival of 45% even counting with 64.1% of patients at Fontaine’s stage III [1]. Our result of 72% is slightly lower than in both studies, but compares well, suggesting that TcpO_2_ and stimulation trials are not mandatory to achieve good clinical outcomes. In our experience, TcpO_2_ measurements are too time-consuming and technically challenging to implement in everyday practice, since the measurements take about 40 min. to complete and require constant room temperature. The outcome may also be influenced by cardiac and pulmonary function, skin thickness, edema, and obesity. Implementing trial stimulation in everyday practice is much more feasible. However, prior to this study, we experienced a severe infection in one patient after such a trial. Subsequently, having observed that PAD patients may have compromised immune systems, we changed our clinical practice and moved to a single-stage implant procedure without trial stimulation.

Our slightly lower probability of limb survival at 12 months compared with the ESES and SCS-EPOS studies may be due to its real-world context. For example, in our study, 70% of patients were classified as Fontaine’s stage IV. This high percentage reflects the fact that patients are not referred for revascularization until quite late in their disease progression. According to the SCS-EPOS study, the percentage of Fontaine’s stage IV patients in the SCS-match group was only 56%.

As with all surgical procedures and long-term treatment with implanted hardware, SCS therapy has associated complications. In the Cochrane Library meta-analysis of SCS for the treatment of non-reconstructable chronic CLI, the pooled risk of implantation problems was 8%, lead dislocation or fracture 12%, and infection 3% [14]. In our study, lead dislocation ocurred at a rate of 4.2% and infection at 1.4%. Despite over one-quarter of our cohort being diabetic, no infections occurred in this subgroup. Our complication rates are below, or within, the published ranges for SCS procedures [2, 8, 10–12].

### Limitations

Interpretation of the outcomes of this study is limited because the study design was not controlled, and data were collected retrospectively. Our results are derived from a patient population of truly refractory patients and add to the growing body of evidence that is broadly supportive of treating selected CLI patients with SCS therapy. However, further randomized controlled studies are required to confirm these findings, as well as to establish the additional benefits of TcpO_2_ measurements and trial stimulation in patient selection.

## Conclusions

Our results suggest that SCS is an appropriate, safe, and effective additional therapy option in non-reconstructable CLI patients selected based on clinical parameters. The therapy may be more beneficial in patients classified as Fontaine’s stage III.

## Data Availability

Data is kept confidential, informed consent was not required for this retrospective study.

## Abbreviations

SCS: Spinal cord stimulation
CLI: Critical limb ischemia
PAD: Peripheral arterial disease
IPG: Implantable pulse generator
TcpO2: Transcutaneous oxygen pressure
